# Observation of Third-order Nonlinearities in Graphene Oxide Film at Telecommunication Wavelengths

**DOI:** 10.1038/s41598-017-09583-6

**Published:** 2017-08-29

**Authors:** Xiaochuan Xu, Xiaorui Zheng, Feng He, Zheng Wang, Harish Subbaraman, Yaguo Wang, Baohua Jia, Ray T. Chen

**Affiliations:** 1 Omega Optics, Inc., 8500 Shoal Creek Blvd., Bldg. 4, Suite 200, Austin, TX 78757 USA; 2 Centre for Micro-Photonics, Faculty of Science, Engineering and Technology, Swinburne University of Technology, P. O. Box 218, Hawthorn, VIC 3122 Australia; 3 Department of Mechanical Engineering, The University of Texas at Austin, Austin, Texas 78712 USA; 4 Materials Science and Engineering Program, Texas Materials Institute, The University of Texas at Austin, Austin, Texas 78712 USA; 5 Department of Electrical and Computer Engineering, Boise State University, Boise, Idaho 83725 USA; 6 Department of Electrical and Computer Engineering, The University of Texas at Austin, 10100 Burnet Rd., MER 160, Austin, Texas 78758 USA

## Abstract

All-optical switches have been considered as a promising solution to overcome the fundamental speed limit of the current electronic switches. However, the lack of a suitable third-order nonlinear material greatly hinders the development of this technology. Here we report the observation of ultrahigh third-order nonlinearity about 0.45 cm^2^/GW in graphene oxide thin films at the telecommunication wavelength region, which is four orders of magnitude higher than that of single crystalline silicon. Besides, graphene oxide is water soluble and thus easy to process due to the existence of oxygen containing groups. These unique properties can potentially significantly advance the performance of all-optical switches.

## Introduction

The concept of using all-optical switch - manipulate photons with photons - to avoid electro-optic conversion in optical communication networks is almost as old as the optical communication itself. Yet only recently has serious attention been given because in the past the innovation and breakthroughs in electronics were able to keep up with the relentless exponential growth of the Internet Protocol (IP) traffic^1, 2^. As the IP traffic continues to grow without foreseeable sign of halting, electronic devices are rapidly approaching their fundamental speed limit. It has been widely accepted that for switching speed above 100 GHz, all-optical switching is the only viable solution^3^.

To be deployed into optical communication networks, all-optical switches must satisfy the requirements in cost, size, weight, and power consumption (C-SWaP). Among all the material platforms that have been intensively investigated, silicon is the most potent candidate. The fabrication maturity of the silicon electronics industry can be leveraged to rapidly and cheaply create a large number of optical devices with incredible economies of scale. While recent breakthroughs in integrated silicon photonics^4–6^ have laid a solid foundation for photonic devices that can fulfill the C-SWaP prerequisites, the lack of a suitable third-order nonlinear material deters the development efforts. Silicon has relatively high third-order nonlinearities and its large refractive index facilitates the tight confinement of light inside the submicron single mode waveguide, leading to an extraordinarily high nonlinear parameter *γ* up to 3 × 10^2^ W^−1^m^−1^ ^7, 8^. *γ* is defined as *n*_2_/(*λA*_*eff*_), where *A*_*eff*_ is the effective area of the waveguide, λ is the wavelength and *n*_2_ the Kerr coefficient^8^. However, materials with large Kerr coefficients usually have small bandgap and therefore also have strong two-photon absorption (TPA) effect, which not only results in the loss of photons but also causes undesired free carrier absorption (FCA) and dispersion (FCD)^9^. The nonlinear figure of merit (FOM), defined as n_2_/(*β* λ), is used to quantize the intrinsic limitation, where *β* is the TPA coefficient. The FOM of silicon is only 0.3. A few materials have been investigated as alternatives such as amorphous silicon and silicon nitride^7, 8^. Although these materials have amazingly large FOM values, they suffer from small Kerr coefficient and hence result in power-hungry devices.

In recent years, there is growing interest in the optical nonlinearities of two-dimensional (2D) materials. Graphene shows a remarkable Kerr coefficient of ~102 cm^2^/GW at 1550 nm^10^, but it is accompanied by highly nonlinear absorption due to the zero bandgap. Thus, Graphene has been intensively studied as a promising saturable absorber^11, 12^. Black Phophorous (BP) has strong saturable absorption, but its Kerr effect is negligible^13, 14^. Few-layer oxidized Black Phosphorous (OBP) has a Kerr coefficient of ~10^−2^ cm^2^/GW^13, 14^. However, its nonlinear absorption is high. Bi_2_Se_3_ shows a nonlinear refractive index of 10 ^−1^ cm^2^/GW at 800 nm^15^, but it is highly absorptive at telecommunication wavelength as its bandgap is ~0.3 eV. Recently, Graphene oxide (GO) has become a rising star in the graphene family due to its unique physical and chemical properties originated from the hybridization of the *sp* ^2^- and *sp* ^3^- carbon atoms. One intriguing property is that its optical and electrical properties can be tuned dynamically by manipulating the content and location of oxygen containing groups through either chemical or physical reduction^16–20^. A recent study on laser reduction unveils the potential of achieving a large nonlinear coefficient and large enough FOM simultaneously^21^. An *n*_2_ of ~15 cm^2^/GW has been observed at 800 nm, which is more than five orders of magnitude larger than that of Silicon, while its FOM is as large as 4.56^21^. Its nonlinear properties are stable under high-power illumination up to 400 mJ/cm^2, 22^. Due to the existence of oxygen containing groups, GO is hydrophilic and water soluble, making it easy to process. Although numerous research activities have been reported, few are in the telecommunication wavelength range. In this paper, we report the ultrahigh third-order nonlinearity of the GO film synthesized with the vacuum filtration-and-transfer technology. The observed *n*_2_ is four orders of magnitude larger than single crystal silicon with negligible high order absorption, which makes GO a promising candidate for all-optical switches in the telecommunication regime.

## Results

**Figure 1:**
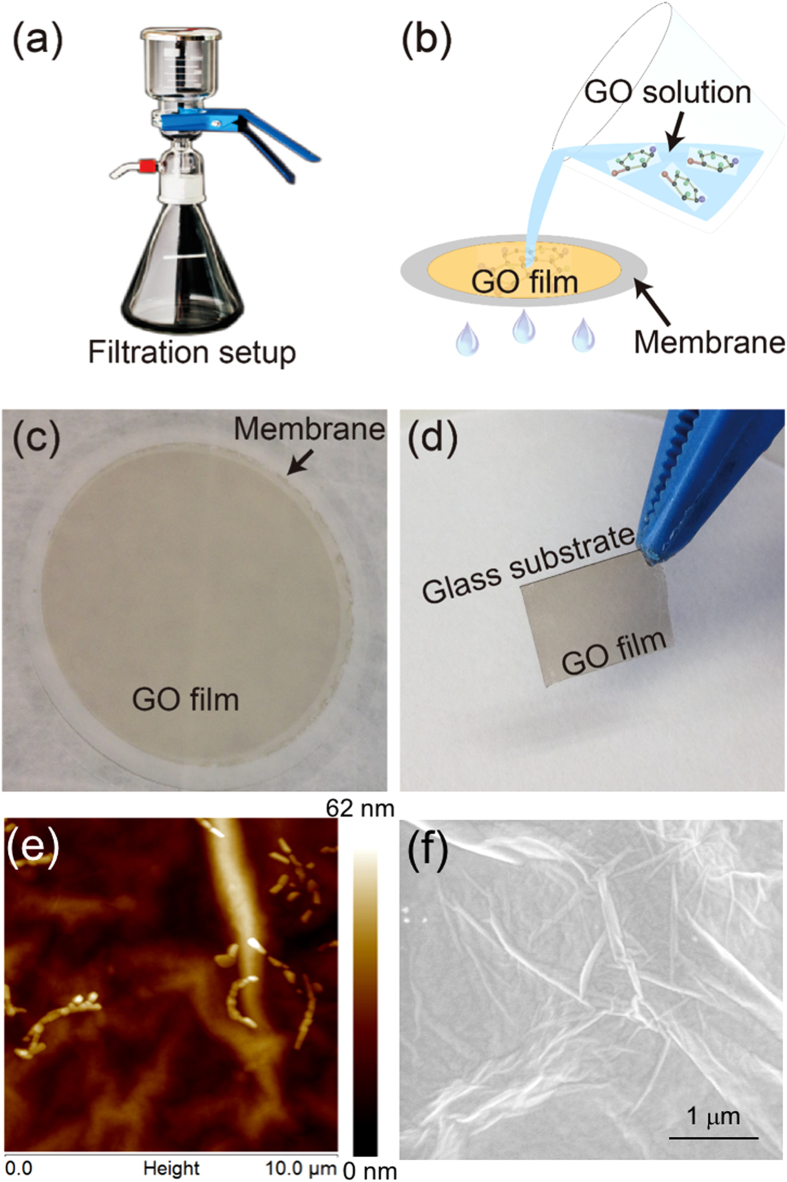
(**a**) The schematic diagram of the filtration setup. (**b**) Schematics of the vacuum filtration process for the GO suspensions. (**c**) The filtrated GO thin films on the AAO membrane. (**d**) The transferred GO thin film on the glass substrate. (**e**) The AFM images and (**f**) the SEM image of the GO thin film on the glass substrate.

The GO films are synthesized by the chemical reduction of graphite via a modified Hummers method^23^. Firstly, graphite and NaNO_3_ are mixed with concentrated H_2_SO_4_. By vigorous stirring, the reducing agent –KMnO_4_ is added into the suspension. Then H_2_O_2_ is added into the mixture at 98 °C. The product is washed and dried, and then the GO sheets are obtained after the purification. For the self-assembly of GO sheets, a deionized water/methanol mixture with an optimal ratio of 1:5 is used to disperse GO. The GO thin films have been synthesized via the vacuum filtration method by using the schematic setup in Fig. 1(a) ^24^. The vacuum filtration process involves the filtration of a GO suspension through an anodic aluminium oxide (AAO) or polyethylene terephthalate (PET) membrane. As the liquid (water) passing through the membrane, the GO sheets are filtered on the membrane, forming the high quality GO thin films with desired thickness, as shown in Fig. 1(b) and Fig. 1(c). Then the filtrated highly uniform GO thin films on the membrane (Fig. 1(c)) can be transferred onto various substrates, such as the glass slide (Fig. 1(d)), by either dissolving the membrane or peeling off the film with the aid of water. Moreover, the nanoscale control over the film thickness can be achieved by simply varying either the concentration of the GO solution or the filtration volume.

The thickness of the GO thin film is 1 μm and is transferred onto a 125 μm thick cover glass slide. Atomic Force Microscope (AFM) measurements show that the surface roughness is around 34 nm, as shown in Fig. 1(e). The scanning electron microscope (SEM) image of the GO thin film is shown in Fig. 1(f). The linear absorption of the GO thin film is measured by the Fourier transform infrared spectroscopy (FTIR). The absorption of GO is weaker at telecommunication wavelength compared to its absorption at the shorter wavelengths due to the smaller photon energy^21^. The Raman spectroscopy of the GO thin film can be found in ref. 21.

**Figure 2:**
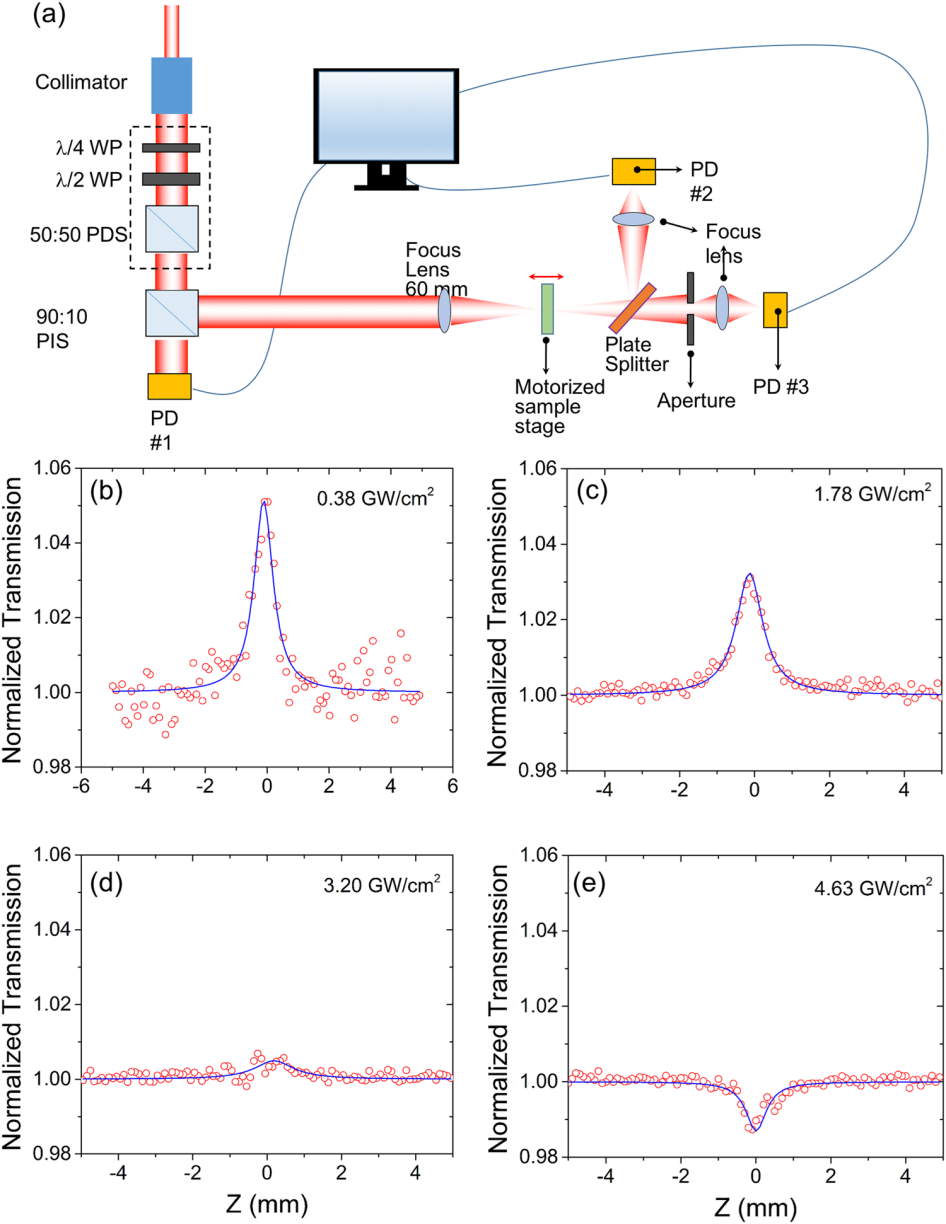
The z-scan system (**a**) and the open aperture z-scan curves when the peak irradiance equals to (**b**) 0.38 GW/cm^2^, (**c**) 1.78 GW/cm^2^, (**d**) 3.20 GW/cm^2^, and (**e**) 4.68 GW/cm^2^. The red circles are experimental data and the blue curves are theoretical fittings. WP - waveplate, PDS - polarization dependent splitter, PIS - polarization independent splitter, PD - photodiode.

The third-order nonlinearity of the GO film at the telecommunication wavelength is measured with the z-scan system shown in Fig. 2(a). Calmar femtosecond laser with a pulse width of 67 *f*s and a repetition rate of 20 MHz is used as the light source. The spectrum of the pulse is ~ 40 nm wide and centers at 1560 nm. Light from the laser is collimated and split into two channels at a ratio of 90:10. The 10% of power is used to monitor the power fluctuation and avoid fake signals^25^. The power of the beam is controlled through a combination of a quarter wave plate, a half wave plate, and a polarization dependent splitter. An *f* = 60 mm bi-convex lens is used to focus the beam. The sample is mounted on a motorized stage. A 50:50 plate beam splitter is used to split the beam into the open and close channels. A Labview program is developed to synchronize the movement of the motorized stage and data collection. Since the surface roughness of the GO is large compared to semiconductor materials, e.g. silicon, a continuous-working (CW) laser is exploited as a low irradiance source for background scattering subtraction^26^. A detailed description of the z-scan system is provided in the method section.

Both open and closed aperture z-scan curves have been taken under irradiances between 0.38 GW/cm^2^ and 4.63 GW/cm^2^. The representative open aperture z-scan curves are shown in Fig. 2(b–e). As GO contains *sp* ^2^ *-* clusters in insulating *sp* ^3^ *-* matrix, its nonlinear behavior is a complicate interplay of *sp* ^2^- and *sp* ^3^- domains^19, 27^. However, since *sp* ^3^- hybridization has an energy gap of 2.7~3.1 eV^19, 27^, which is larger than the energy of a single photon at the wavelength of 1.55 μm, the *sp* ^2^- hybridization plays a dominant role in forming the open aperture z-scan curve. The size of the localized *sp* ^2^- clusters determines the energy gap, and GO usually contains a range of different sizes. Therefore, the collective band structure has no signature features^17, 27^. The existence of 2~3 nm *sp* ^2^- clusters has been confirmed with multiple metrologies, which correspond to an energy gap of ~0.5 eV^17, 21, 27–29^. The optical absorption of electrons can be easily saturated, resulting in the depletion of the valence band^21^. As shown in Fig. 2(b), at an irradiance *I*_0_ of 0.38 GW/cm^2^, prominent saturable absorption (SA) is observed. As the irradiance increases to 1.78 GW/cm^2^, the SA peak decreases, as shown in Fig. 2(c), suggesting the increment of the saturable intensity (*I*_*s*_), which is an indication of the increase of *sp* ^2^ *-* and *sp* ^3^ *-* ratio induced by the reduction of GO to rGO. The reduction of GO creates new *sp* ^2^ *-* clusters, through removal of oxygen. The process could provide percolation pathways among the existing *sp* ^2^ *-* domains^17^. When *I*_0 _ = 3.20 GW/cm^2^, SA becomes negligible due to the ratio of *sp* ^2^ *-* and *sp* ^3^ *-* has reached a value that the electrons on the ground states cannot be further depleted, as shown in Fig. 2(d). When *I*_0_ continues to increase, reverse saturable absorption (RSA) has been observed, as shown in Fig. 2(e), which is possibly due to the excited state absorption (ESA).

**Figure 3:**
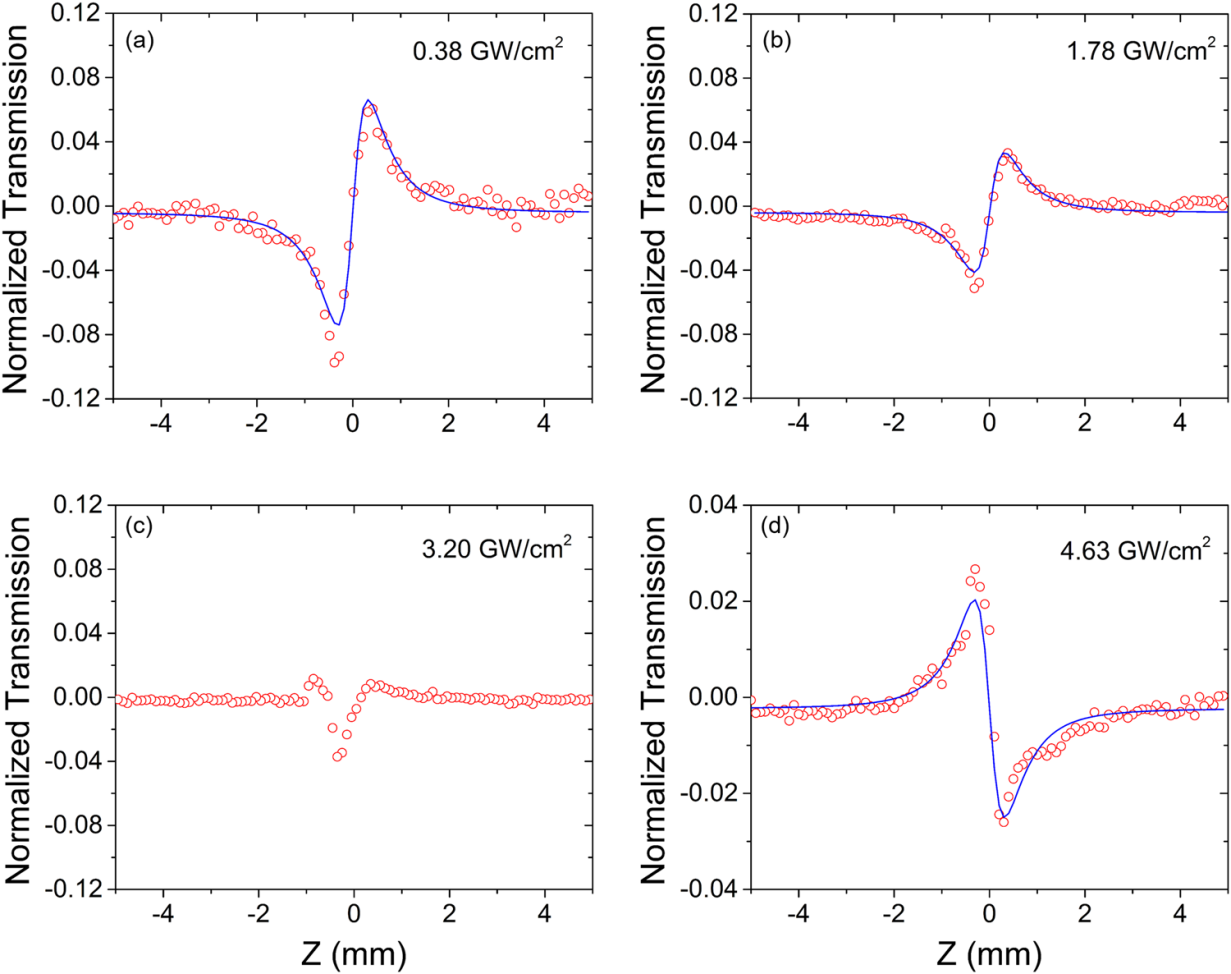
The close aperture z-scan results under different irradiances: (**a**) 0.38 GW/cm^2^; (**b**) 1.78 GW/cm^2^; (**c**) 3.20 GW/cm^2^; (**d**) 4.68 GW/cm^2^. The red circles are experimental data and the blue curves are theoretical fittings.

The closed aperture z-scan curves are shown in Fig. 3. As aforementioned, a low irradiance z-scan curve is measured as a reference to subtract the scattering induced fake signals^25^. The Iris aperture was set to 0.3. When *I*_0_ = 0.38 GW/cm^2^, paramount self-focusing was observed, as shown in Fig. 3(a). The peak-valley modulation exceeds 16%. The observed nonlinear behavior is primarily due to the population redistribution of the *π* electrons, the free carriers of the *sp* ^2^- domain, and the bounded electrons and free carriers of the *sp* ^3^- matrix^19^. Due to the relatively high repetition rate of laser pulses, the z-scan process is accompanied by the reduction of GO. As a result, the closed aperture curve is asymmetric. The *n*_2_ is estimated to be 0.45 cm^2^/GW, which is four orders of magnitude higher than that of single crystal silicon^8^. With a lower peak irradiance or a smaller repetition rate, a stronger and symmetric closed aperture curve can be observed. However, due to the detection limitation of our in-house z-scan system, it is difficult to observe the nonlinear response with low peak irradiance. When *I*_0_ increases to 1.78 GW/cm^2^, the self-focusing effects are abated, which is believed to be induced by the reduction of GO. Subsequently, the *n*_2_ decreases to 0.13 cm^2^/GW, yet it is still a few orders of magnitude larger than that of the single crystal silicon^26^. When the peak irradiance increases to 3.20 GW/cm^2^, the transition from valley-peak to peak-valley (self-defocusing) configuration occurs. The z-scan curve is very difficult to fit due to the co-existence of two distinct nonlinear responses. When *I*_0_ further increases to 4.63 GW/cm^2^, the closed aperture z-scan curve changes to peak-valley configuration, indicating a negative *n*_2_, which is a confirmation of the existence of the reduction and the change of *sp* ^2^-*/sp* ^3^- ratio. The *n*_2_ is estimated to be −0.012 cm^2^/GW. The observed behavior is consistent with the previously reported nonlinear properties of graphene^10, 21^. Thus the nonlinear refractive index is mainly attributed to the *sp* ^2^- clusters after reduction.

**Figure 4:**
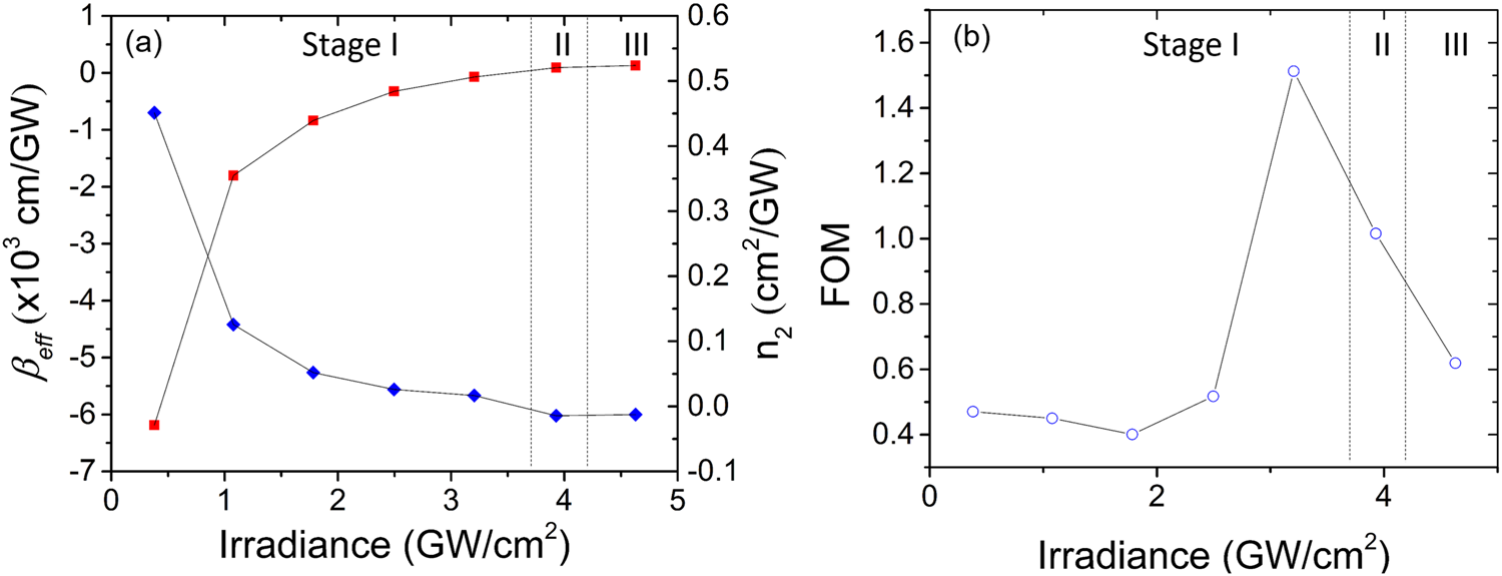
(**a**) The *n*2 (red) and equivalent coefficient βeff (blue) in relation to the peak irradiance *I*0. (**b**) FOM under different peak irradiance.

Figure 4a summarizes the magnitude of the nonlinear responses of the GO thin film under different irradiances. Both the nonlinear refractive index *n*_2_ and the nonlinear absorption coefficient *β* as a function of the irradiance *I*_0_ are deduced by fitting the closed aperture and open aperture z-scan curves, respectively. For open aperture fittings, *β*_eff_ is used to account the combined effects of SA and RSA^19^. Thus the FOM is defined as n_2_/(*βeffλ*) which is shown in Fig. 4b. The entire process can be separated into three stages. In stage I, *n*_2_ is positive and *β*_eff_ is negative. SA dominants the overall nonlinear response in the open channel. For peak irradiance less than 3 GW/cm^2^, the FOM is around 0.5. As the peak irradiance *I*_0_ continues to increase, the absolute values of both *n*_2_ and *β*_eff_ decrease. The absolute value of FOM increases significantly to approximately 1.5. Stage II is the transition stage, in which the value of *n*_2_ is difficult to estimate due to the co-existence of the self-focusing and the self-defocusing. The z-scan closed aperture curve does not show clear peak-valley or valley-peak configurations either. In Stage III, both *n*_2_ and *β*_eff_ change signs, indicating that the majority of *sp* ^3^- regions have been reduced.

## Discussion

In Stage I, although the absolute value of FOM is close to that of silicon, *β*_eff_ is negative, which indicates a prominent SA. One should note that *β*_eff_ is an equivalent coefficient which combines the effects of SA and other nonlinear absorptions. Thus, the FOM cannot be reliably calculated with the formula provided in the introduction section. Since the majority of *sp* ^2^- clusters are around 3 nm, corresponding to a band gap around 0.5 eV, and the energy gap of the *sp* ^3^- hybridization is 2.7~3.1 eV, figure of merit (FOM), defined as n the nonlinear absorption in stage I is negligible. Thus, although its FOM is close to silicon in Stage I, GO is a very promising material for all-optical switches. When the peak irradiance increases to beyond 3 GW/cm^2^, the FOM increases significantly to ~1.5. However, *n*_2_ decreases to 1.67 × 10^−2^ cm^2^/GW, more than one order of magnitude smaller than the value before reduction. In the transition stage (Stage II), due to the coexistence and competition of two distinguish nonlinear effects, there is no clear overall nonlinear effect observed in the closed aperture z-scan curve, and therefore GO in this stage is not suitable for all-optical switch applications. In Stage III, the FOM is similar as that of the Stage I. *β*_eff_ is positive and a clear RSA is observed in the open channel z-scan curve. *n*_2_ is small in this stage. Further reduction of the GO can possibly increase *n*_2_, but the nonlinear absorption will increase simultaneously. Based on the analysis, GO in stage I is most promising for all-optical switching applications. Table 1 summarizes the nonlinear coefficients of materials compatible with semiconductor fabrication process. The n_2_ of GO is four orders of magnitude larger than c-Si.

**Table 1:** Nonlinear Optical Materials.

	a-Si^30^	c-Si^7^	SiN^31^	Graphene^10^	GO^21^	rGO^21^	GO
Wavelength	1550 nm	1550 nm	1550 nm	1550 nm	800 nm	800 nm	1550 nm
n_2_ (cm^2^GW^−1^)	1.82 × 10^−4^	4.55 × 10^−5^	2.6 × 10^−6^	10^2^	7.5	~15	0.45

To further improve the performance of GO for all-optical switching applications, *sp* ^3^-/*sp* ^2^- ratio should be further increased. Since the close channel z-scan curve changes from valley-peak to peak-valley configuration during the reduction process, the positive nonlinear coefficient roots in the *sp* ^3^- hybridization and negative in the *sp* ^2^-. Increasing *sp* ^3^-/*sp* ^2^- ratio not only increases the nonlinear effect but also reduces the linear absorption and nonlinear absorption. Besides, the *sp* ^2^- induced nonlinear refractive index is from carrier effects, the relaxation time of which is tens of picoseconds due to the bandgap of the *sp* ^2^- hybridization^20^. To achieve faster switching speed, *sp* ^3^- hybridization is highly preferred.

In summary, we investigated the third-order nonlinear properties of the GO film at 1550 nm wavelength range with a z-scan set-up. The results show an exceptionally high *n*_2_, which is about four orders of magnitude larger than single crystalline silicon. In the meantime, the nonlinear absorption is negligible due to the large energy gap of the *sp* ^3^- hybridization. Due to the existence of oxygen containing groups, GO is hydrophilic and water soluble. Thus it can be easily applied as the cladding of existing all-optical switch structures, such as ring resonators, slot waveguides and photonic crystal cavity. These properties make GO a potent candidate for all-optical switches.

## Methods

### Z-Scan

Figure 2(a) shows the z-scan system built. The laser beam is collimated through a 7 mm collimator (Thorlab, F810FX-1550). A quarter wave plate (QWP, Thorlab WPG10M-1550) converts circular polarization into linear polarization. A half-wave plate (HWP, Thorlab WPH10M-1550) is used to rotate the linear polarization direction^32^. Together with the 50:50 polarization dependent splitter cube (PDS, Thorlab CM1-PBS254), the quarter wave plate is used as a tunable attenuator to control the irradiance. Another 90:10 polarization independent splitter (PIS, Thorlab BS030) is used to split 10% of the total power for system monitoring to avoid the fake nonlinear signals^25^. An *f* = 60 mm bi-convex lens (Thorlab LB1723-C) is used to focus the light beam. The sample is mounted on a motorized stage (Thorlab MTS25-Z8E). A 50:50 plate beam splitter (PBS, Thorlab BSW12R) is used to split the beam into two channels, open and close. In the open channel, an *f* = 60 mm bi-convex lens (Thorlab LB1723-C) is used to ensure the collection of all the power in this channel. A photodiode (PD#2 Newport 818IR) is exploited to monitor the power. To ensure the photodiode works at the linear response range, a 10 × attenuator is mounted in front of PD#2. In the close arm, an Iris aperture and another *f* = 60 mm bi-convex lens (Thorlab LB1723-C) are used to harvest all the light passing through the aperture. PD #3 is used to detect the power in the close arm. The signals from PD #1~3 are collected with a BNC board (National Instrument BNC2110) and PCI card (National Instrument PCIe-6321). A Labview program is developed to synchronize the movement of the motorized stage and data collection. Calmar femtosecond laser with a pulse width of 67 fs and a repetition rate of 20 MHz is used as the light source. The spectrum of the pulse is ~40 nm wide and centered at 1560 nm. Since the surface roughness of the GO is large compared to semiconductor materials, e.g. silicon, a continuous-working (CW) laser is exploited as a low irradiance source for background scattering subtraction^26^.
